# Simultaneous Metastasis from Cervical Cancer to the Kidney and Paraspinal Muscle: A Case Report

**DOI:** 10.7759/cureus.4148

**Published:** 2019-02-27

**Authors:** Juliana Rodriguez, Juan C Castro, María Beltran, Oscar Forero, Rene Pareja

**Affiliations:** 1 Gynecologic Oncology, Instituto Nacional De Cancerologia, Universidad Militar Nueva Granada, Bogotá, COL; 2 Pathology, Instituto Nacional De Cancerologia, Universidad Militar Nueva Granada, Bogotá, COL; 3 Radiology, Instituto Nacional De Cancerologia, Universidad Militar Nueva Granada, Bogotá, COL

**Keywords:** uterine cervical neoplasms, neoplasm metastasis, recurrence

## Abstract

Metastases of squamous cell carcinoma of the cervix to atypical locations may occur in approximately 12% of patients diagnosed with distant metastases, with the kidney and paraspinal muscle as one of the rarest sites of spread.

A 34-year-old woman with a diagnosis of squamous cell carcinoma of the cervix stage IIIB, treated with chemotherapy and radiation, presented 21 months after completion of therapy, with two sites of simultaneous metastases (kidney and paraspinal muscle). No other evidence of disease was noted. She underwent right nephrectomy and radiotherapy to the para-spinal mass. She did not accept chemotherapy. The patient then had progression of disease in the right nephrectomy bed and a new left renal lesion. The paraspinal lesion presents a partial response. The patient declined further chemotherapy and died five months after the relapse.

Simultaneous metastases of squamous cell carcinoma of the cervix to the kidney and paraspinal region is a rare entity, and there is currently no standard recommendation for treatment.

## Introduction

Cervical cancer is the fourth most common cancer in women worldwide, with 527,600 new cases every year, 265,700 deaths, and 85% of cases occurring in developing countries [1]. Cervical cancer spread occurs primarily by contiguity, however, it may also spread through lymphatic channels and regional lymph nodes. Less frequently, a hematogenous spread is noted. The latter is responsible for metastases to the lung (26.5%), liver (15.8%), bone (14.2%), bowel (8.2%), adrenal glands (3.8%), spleen (2.3%), or brain (1.4%) [2].

Among the unusual metastases of cervical cancer, renal metastasis is an infrequent presentation, with 13 cases previously reported in the literature [3]. The incidence of skeletal muscle metastasis is <1% of all hematogenous dissemination and since 2008, only a few cases have been published [4]. The prognosis of patients with metastatic cervical cancer is poor and the options for systemic treatment are limited [5].

There is a paucity in the literature regarding management strategies for this rare presentation of metastatic cervical cancer. Our aim is to present a case of simultaneous unusual metastases to the kidney and paraspinal muscle and to review the current literature for helping shed light on potential options of therapy.

## Case presentation

A 34-year-old woman was diagnosed with a non-keratinizing, moderately differentiated, large cell squamous cell carcinoma of the cervix, stage IIIB, in 2015. She underwent concomitant chemotherapy (paclitaxel 90 mg/m^2^ plus carboplatin 160 mg/m^2^ for six cycles) plus pelvic radiation therapy (5000 cGy) in 25 fractions of 200 cGy). The rationale for the use of such a chemotherapy regimen was not provided by the referring physician. The treatment was completed in July 2015. The patient did not receive brachytherapy immediately, as she was lost to follow-up.

**Figure 1:**
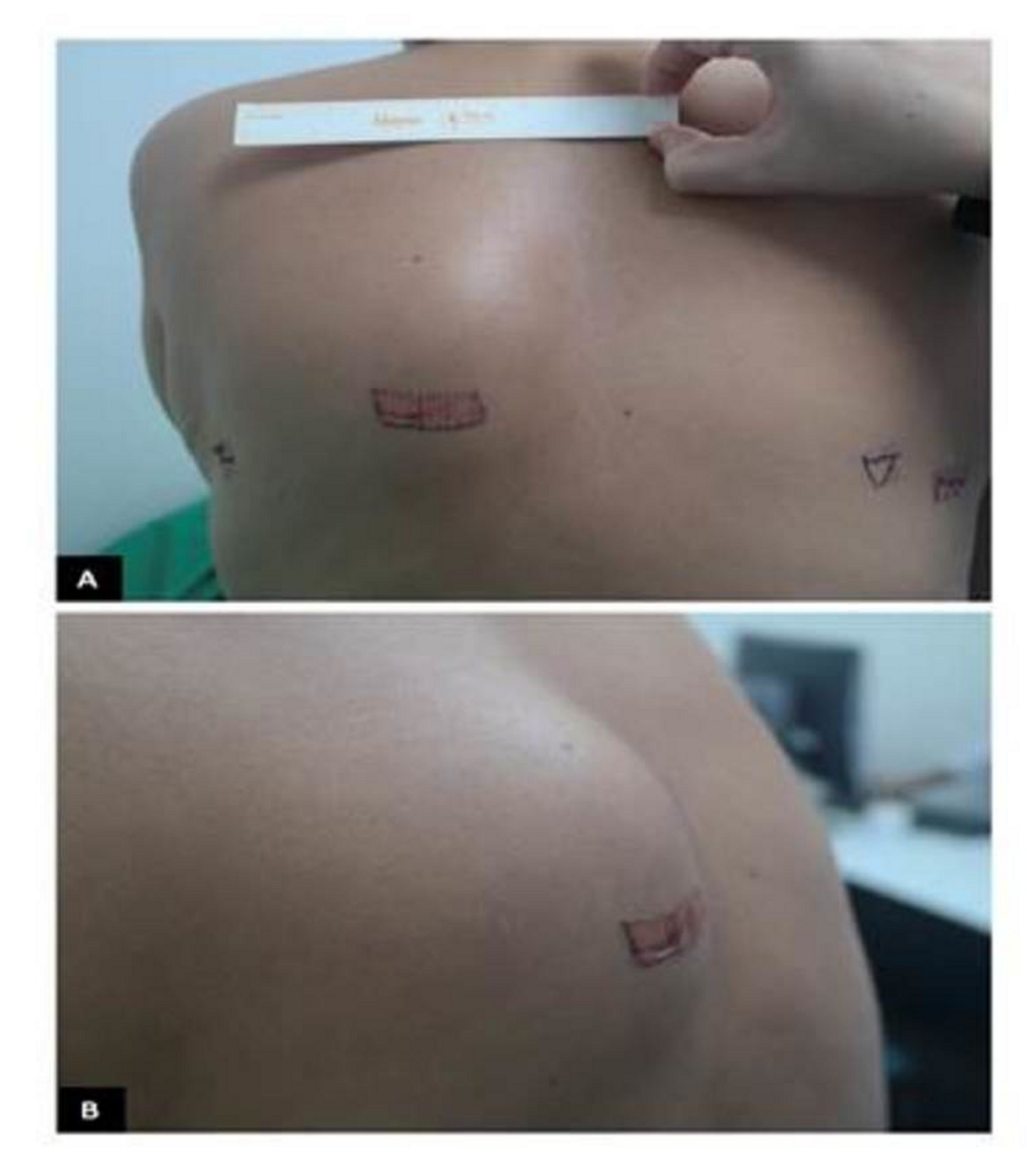
Paraspinal Mass Mass in paraspinal region

She was referred to the Instituto Nacional de Cancerologia 11 months after finishing pelvic radiotherapy for consideration of brachytherapy. In the evaluation, without evidence of cancer, the patient exhibited a grade III rectal toxicity (mucoid, watery diarrhea, more than eight episodes a day). Based on the length of time since the completion of prior therapy and the residual toxicity from prior therapy, it was determined not to administer brachytherapy. The patient was followed without any evidence of recurrent disease; however, 21 months after the completion of therapy, she complained of occasional hematuria without any other symptoms. She also reported a painful inter-scapular mass that was progressively growing over the course of the prior three months. Physical examination showed a solid left upper paraspinal mass, firmly attached to the deep planes, with a diameter of 4 cm (Figures 1A-1B). Pelvic examination showed no evidence of tumor relapse.

**Figure 2:**
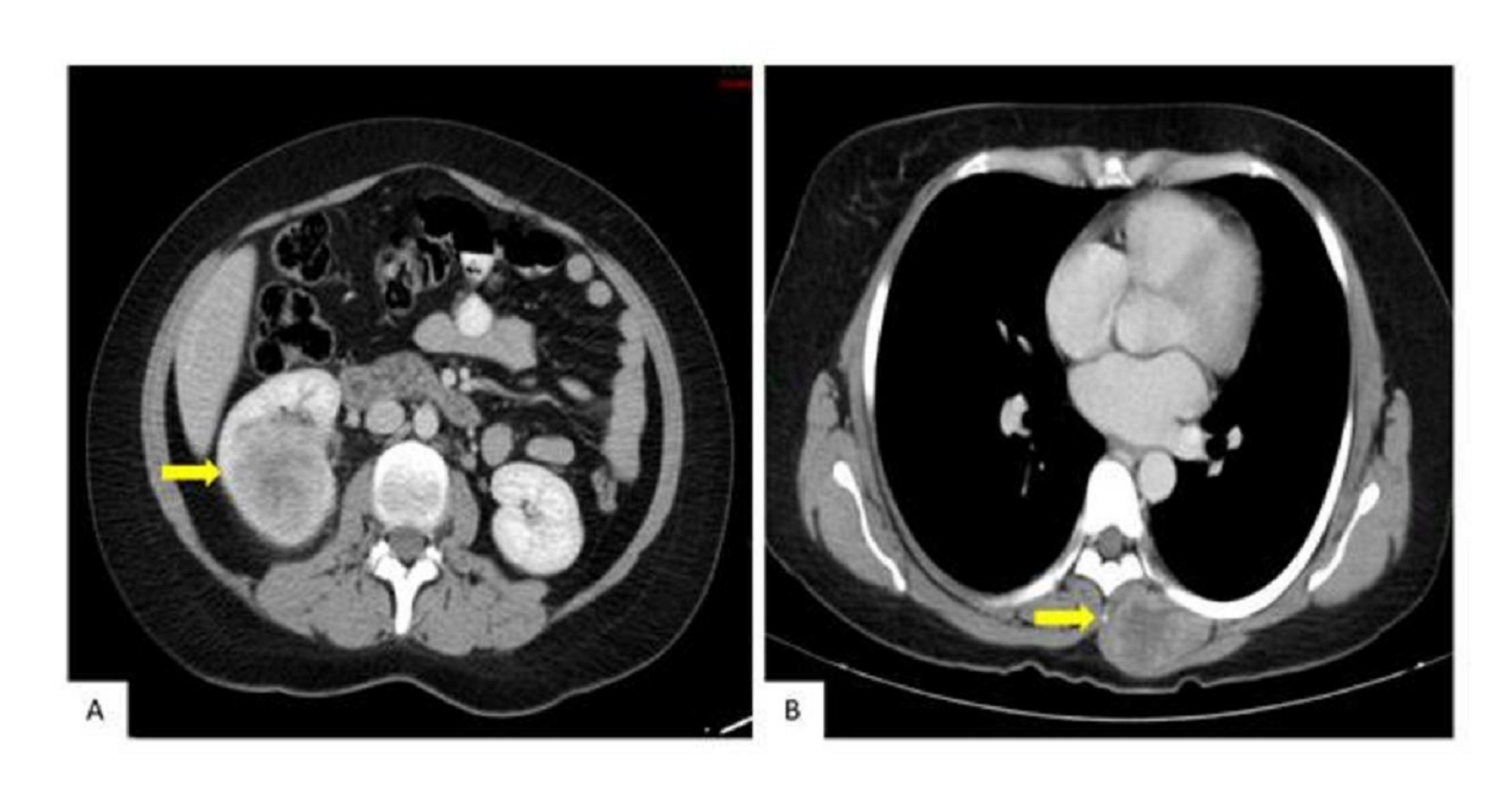
Abdominal and Thoracic CT Scan A: Axial computed tomography (CT) scan of the abdomen showing a predominantly heterogeneous right renal mass with areas of necrosis. B: Thoracic CT scan in axial section showing a mass in the left paraspinal musculature, with signs of necrosis.

An abdominal and pelvic computed tomography (CT) scan showed a solid right renal lesion on the cortex of the middle third and lower pole of the right kidney measuring 4.9x5.1x5.2 cms (Figure 2A). A CT scan of the chest showed a solid lesion with peripheral uptake in the left paravertebral muscles at the level of T5-T8, measuring 3.8x2.8 cms in diameter (Figure 2B).

**Figure 3:**
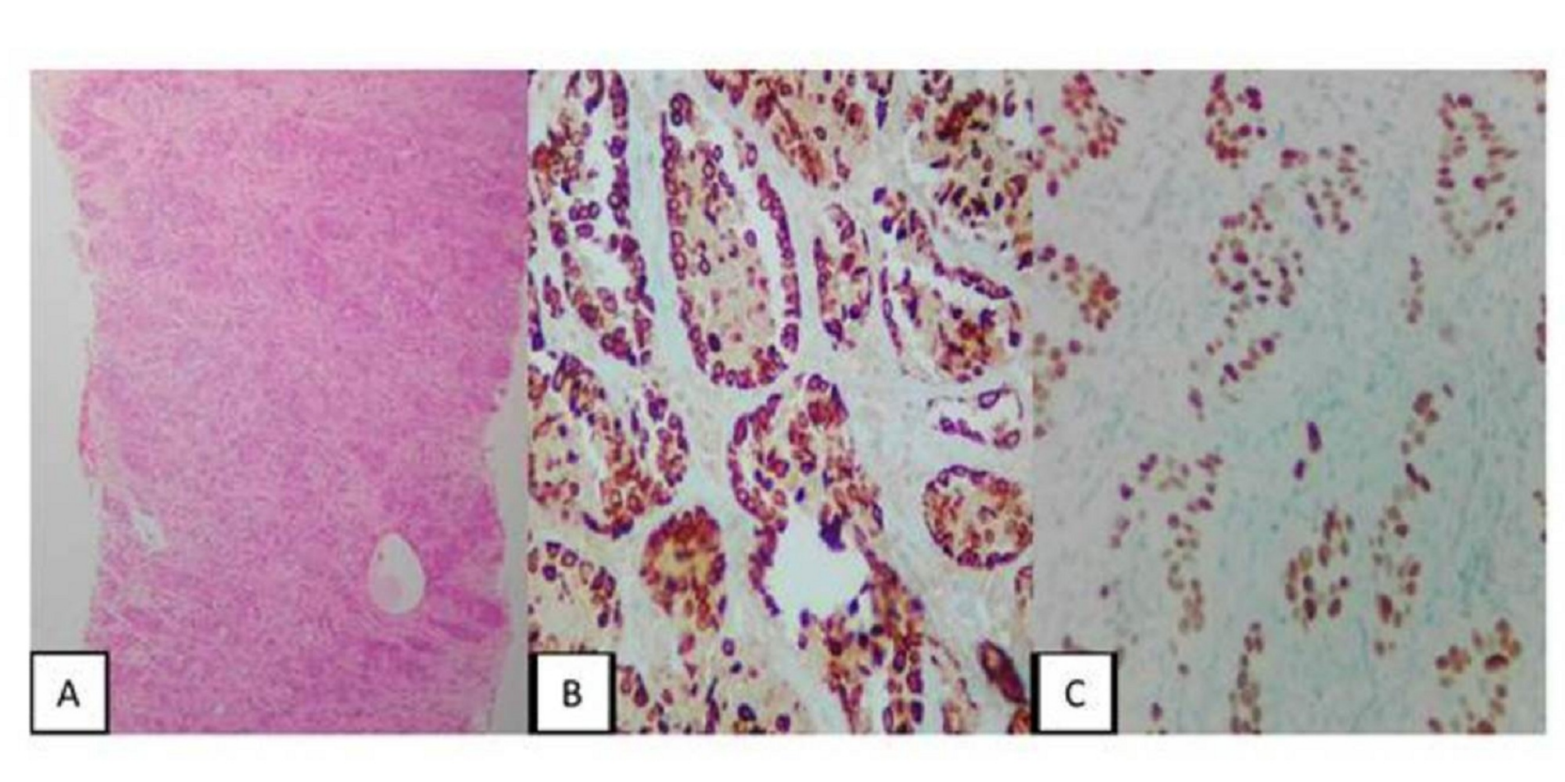
Fine Needle Aspiration Biopsy of the Paravertebral Mass, Immunohistochemistry A: 10X Fibromuscular tissue compromised by poorly differentiated large cell carcinoma. B: Immunohistochemistry (IMH) 40X. Cytokeratin (CK) 5/6 positive for tumor cells. C: IMH 40X. P63 positive for tumor cells. The profile favors squamous cell carcinoma.

A fine needle aspiration biopsy of the paravertebral mass confirmed metastatic, poorly differentiated large cell carcinoma with necrosis. Immunohistochemistry showed a positive immunophenotype for cytokeratin (CK) 7, CK5/6, p63, and p16, favoring squamous cell carcinoma (Figures 3A-3C).

Given the fact that the images showed evidence of extensive tissue infiltration by the paraspinal lesion, it was deemed that surgery would not be ideal.

**Figure 4:**
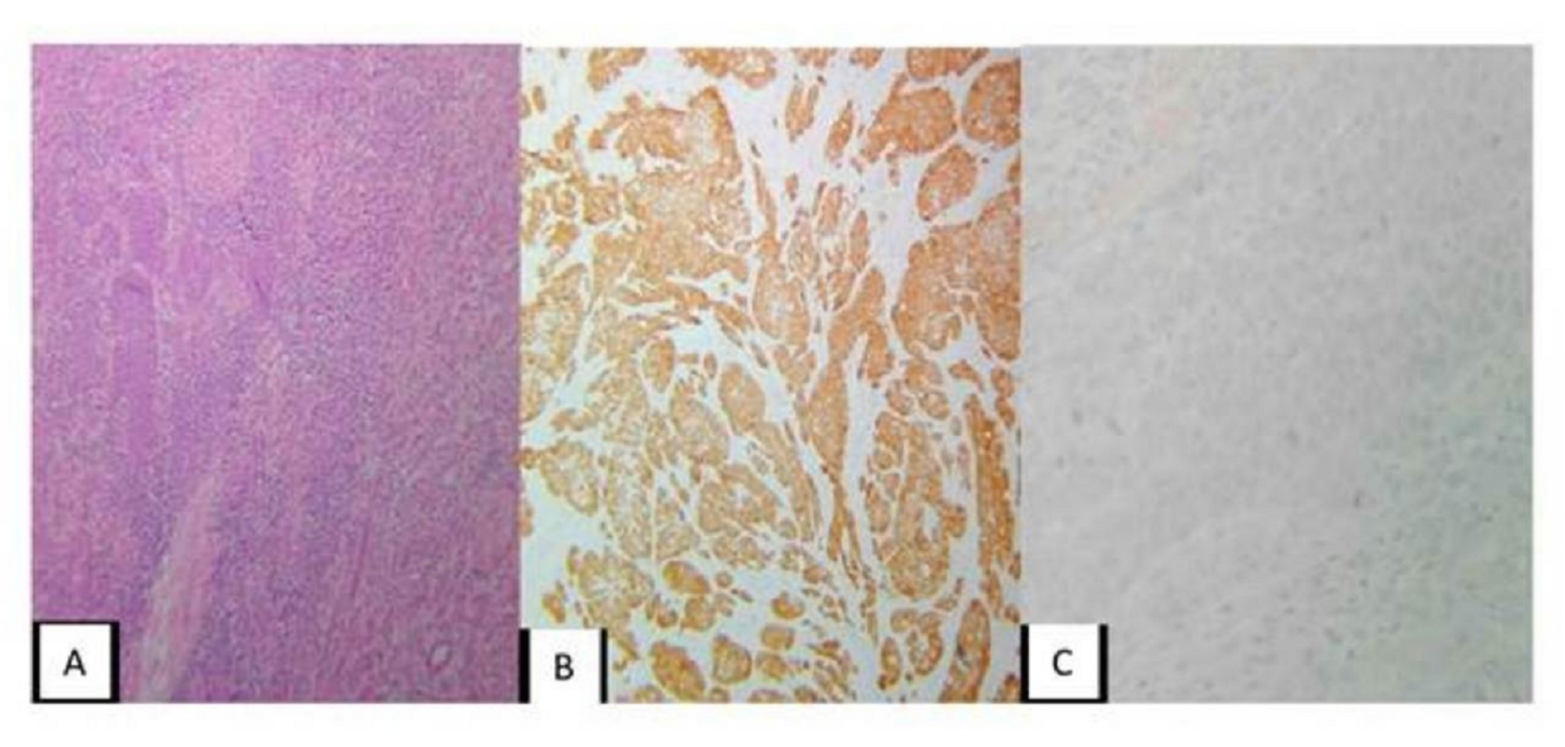
Right Kidney Mass A: Hematoxylin & Eosin (H-E) 10X renal tumor involvement due to squamous, multifocal carcinoma. B: Immunohistochemistry (IMH). 10X P16 positive for tumor cells. C: IMH. 10X RCC negative. The profile favors the metastatic origin of the carcinoma, primary uterine cervix.

The patient underwent a right total nephrectomy by laparoscopy. The pathology report was consistent with metastatic cervix cancer. The immunohistochemistry profile revealed the following: CK AE1/AE3 (+), CK 7 (+), CK 20 (-), p63 (+), CK 5/6 (+), renal cell carcinoma marker (RCC) (-), cell membrane metallopeptidase 10 (CD10) (-), p16 (+), transcription factor protein 3 (guanine - adenine - thymine) (GATA 3): non-contributory (Figures 4A-4C).

Because she had a complete resection of the kidney, without residual lesion, pelvic radiation therapy was not considered. Then, it was decided to give radiotherapy to the para-spinal mass and chemotherapy. She received conformal three-dimensional conformal radiotherapy (3DCRT) radiotherapy to the para-spinal mass, using a fractionation of 300 cGy to complete 3000 cGy. She did not accept receiving chemotherapy.

**Figure 5:**
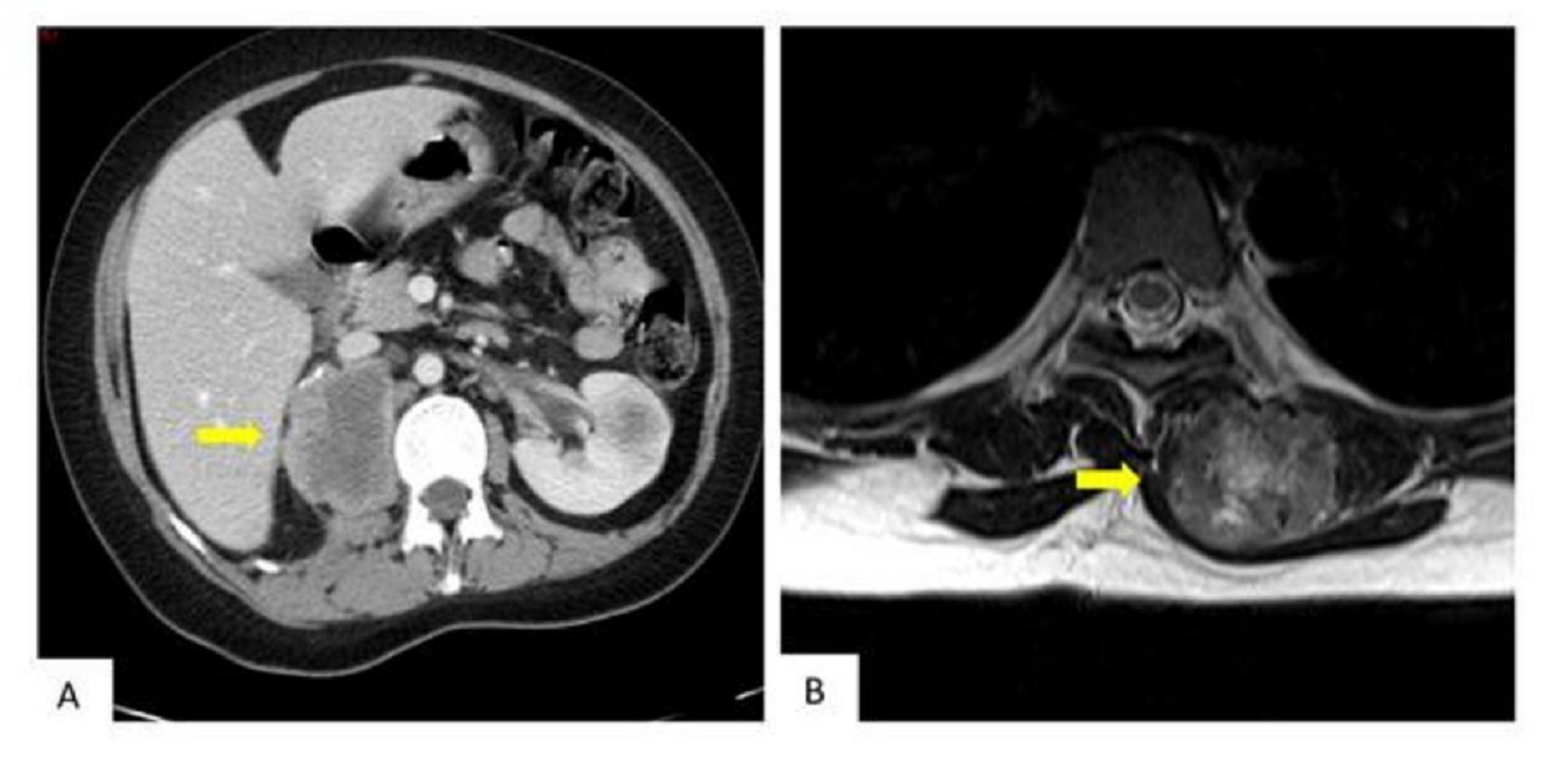
Follow-up Images (Abdominal and Pelvic CT Scan/MRI) A: Computed tomography (CT) of the abdomen in axial section six months after the initial, showing tumor relapse in the right nephrectomy bed and new left renal lesion similar to that documented in the right kidney. B: Magnetic resonance imaging (MRI) in T2 sequence showing the left paraspinal lesion in progression.

Abdominal and pelvic scans in January 2018 showed the progression of the tumor, disease in the right nephrectomy bed, and a new left renal lesion (Figure 5A). Magnetic resonance imaging (MRI) showed the persistence of the paraspinal lesion (Figure 5B). The clinically presented growth of the paraspinal mass was 15x11 cm. The patient declined further therapy and died of the disease in June 2018.

## Discussion

The risk of loco-regional failure, with or without accompanying distant recurrence in patients with locally advanced cervical cancer, can range between 30% and 70% [6]. The average time to recurrence ranges between 7 and 36 months after primary treatment [7]. The addition of bevacizumab to combination chemotherapy in patients with recurrent, persistent, or metastatic cervical cancer was associated with an improvement of 3.7 months in median overall survival (17.0 months vs. 13.3 months) [8]. Hematogenous spread is rare; however, when it occurs, it is primarily found in the lungs, liver, or brain [2]. Kim et al. [9] found that when recurrence presents only as lymphatic metastases, survival is 5.3 times better than when disease recurrence presents by hematogenous metastases. In addition, the completion of standard treatment increases the overall survival by 3.2 as compared to the failure of completion of therapy.

Bhandari et al. [10] found that of 306 patients diagnosed with cervical cancer (3.5% were International Federation of Gynecology and Obstetrics (FIGO) stage IB, 8.5% stage IIA, 9.8% stage IIB, 67% stage IIIB, and 11% stage IVA), 8.17% developed distant metastases. Of these, 12% corresponded to metastases of an unusual location, one in the breast, one in the paraspinal muscle, and one in the duodenum.

Skenderi et al. described 19 cases of metastasis from cervical cancer to skeletal muscle [4]. In reported cases, the predominant histological type is squamous cell carcinoma and most patients had a locally advanced disease [4,11]. This metastasis was found at the time of diagnosis of cervical cancer in some patients, however, it can occur at any time during the post-treatment follow-up, up to 180 months [4,11]. The most common symptom is a painful mass in a muscular area and the most frequently diagnosed site is the psoas muscle [4]. A biopsy should always be performed to confirm the diagnosis. The prognosis is unfavorable because there is no consensus about the treatment; this indicates that a multi-modal approach is often the best option. Ideally, surgical resection should be performed, along with systemic therapy with chemotherapy [11]. If it is not possible, radiotherapy alone, or in combination with chemotherapy, provides pain relief and a potential decrease in the size of the metastatic lesion [12].

Metastatic cervical cancer to the kidney is also a rare entity. Bracken et al. [13] found metastatic renal involvement in 7.2% of 11,328 autopsies of patients with any diagnosis of cancer. Zhou et al. [14] described 151 patients with renal metastasis, with the most common primary sites of disease being the lung (43.7%), colorectal area (10.6%), head and neck (6%), breast (5.3%), soft tissues (5.3%), and thyroid (5.3%). There are few cases of cervical cancer with metastases to the kidney [3].

Most patients with metastases to the kidney are asymptomatic, but up to 30% have evidence of hematuria. Most commonly, the diagnosis is based on incidental findings at the time of routine imaging [3]. Less commonly, such metastases may present as a renal mass and fever, suggesting a para-renal abscess [15]. Based on the limited published literature, survival is improved when single isolated lesions are offered surgical resection (partial or radical nephrectomy). The prognosis of patients with metastatic cervical cancer to the kidney is unfavorable, with a median survival of 13 months from the time of diagnosis of metastatic disease [14]. To our knowledge, this is the first reported case of unusual simultaneous metastases to the kidney and the paraspinal muscle region in a patient with locally advanced cervical cancer.

## Conclusions

Simultaneous metastases of squamous cell carcinoma of the cervix to the kidney and paraspinal region is a rare entity, and there is currently no standard recommendation for treatment. To our knowledge, this is the first reported case of unusual simultaneous metastases to the kidney and the paraspinal muscle region in a patient with locally advanced cervical cancer. In this case, we think that several factors could have influenced this kind of unusual relapse, such as receiving unconventional oncological management (she received carboplatin and paclitaxel in concomitance with radiotherapy and did not receive brachytherapy) not in agreement with the primary oncological management described in the literature. The patient also had difficult access to health services and did not fully accept the management options, which limit her timely treatment and diagnosis. She also arrived at our hospital almost one year after having completed her primary oncological management, and we do not know why she received that initial treatment. It is also important to mention that this shows us another form of cervical cancer behavior and that its mechanisms of metastasis are not limited to the lymphatic pathway. Care must be taken to suspect remote commitment in the presence of unusual symptoms and that it could indicate relapses of this type of cancer.
